# Book Reviews

**Published:** 1872-11-01

**Authors:** 


### Book Reviews.

Epidemic Cerebro-Spinal Meningitis, with an appendix on some points on the causes of the disease, as shown by the history of the present epidemic in the city of New York. By Meredith Clymer, M. D. Philadelphia: Lindsay & Blakiston. Chicago: Jansen, McClurg & Co. Price, $1.00.

This is a small monograph of 60 pages, reprinted from the author's additions to the American edition of Dr. Aitken's "Practice of Medicine." From the statistics of the Health Department of New York city, the author has compiled a short history of the present prevailing epidemic in that city, with a view to throw some light on the vexed question of the causes of the disorder. The following quotations embody the main facts evolved from the history.

Cerebro-spinal Meningitis made its appearance as an epidemic in the city of New York, for the first time, about the beginning of the present year (1872). The first series of recognized cases were reported to the Bureau of Sanitary Inspection of the Health Department on the 6th of January. There is no doubt that isolated cases had occurred during the previous year. From January 6 to May 31, inclusive, *six-hundred and thirty-two* (632) cases have been reported to the City Sanitary Inspector, and *four-hundred and sixty-nine* (469) deaths to the Bureau of Records of Vital Statistics. This does not include all the cases happening during that time, for at the beginning of the epidemic, many physicians, from want of personal familiarity with the disorder, failed to make a correct diagnosis, and neglected to report their cases.

At the time of the outbreak of the epidemic there had been a long drought, and the rain-fall had been less during the last three months of 1871, than in the same period of time for some years. The streets and avenues were in a filthy condition; in all, but particularly in those wards which became the chief nests of cerebro-spinal meningitis, there were large collections of garbage, and, necessarily, a great deal of vegetable and animal decomposition. A peculiar and disagreeable odor and taste in the Croton water during the winter and spring, had been noted and commented on. An increase in the common zymotic diseases had been reported to the Health Department. The long-lasting drought had been favorable to the contamination of the springs and wells by the products of vegetable and animal decomposition. The continued absence of rain in the city favored the exhalation of noxious gasses from the heaps of decaying vegetable and animal matter, promoted evaporation from the deep soil, and hindered the proper flushing of the sewers. Reference to the accompanying map, so carefully prepared by Dr. Moreau Morris, City Sanitary Inspector, — and which he has most generously permitted to be engraved for and published with this essay, — will show that the chief nests of the epidemic were in those districts in which the original water courses had been dammed by the filling up of streets and avenues, and over the adjoining water-saturated land. Most of the cases of the disease were grouped on and about the made land, originally marshes. One of the most striking examples is in Collect Place, a swampy spot in the Fourth Ward. Basements over these marshy places were never dry, and the walls are often covered with cryptograms.

In those districts where these factors were absent, the elected haunts were densely populated tenement dwellings, in which the house drainage was invariably found in bad condition. The part played by the diffusion of foul sewer gases in favoring the development of the disease would seem, by the collected evidence, to be unquestionable.

Dr. Morris writes: "Wherever we have carefully examined the local conditions, it has been found that the drainage of the premises has been faulty, or that the immediate surroundings have presented such conditions as must necessarily give rise to some form of disease, — cellars containing decomposed or decomposing vegetables, garbage, or other filth, in a putrefactive condition, and privy vaults located beneath the sleeping-rooms, windows in cul-de-sacs, where there were no free currents of air. The most usual defects discovered were connected with house drainage. These cases are not confined to the abodes of the dirty, squalid, and poor, but houses of a better class, with brown-stone fronts, have furnished their victims. There can be no doubt that overcrowding, with its attendant evils, accumulations of ordure, refuse, and various kinds of filth, absence of a proper supply of pure, fresh air, and personal neglect, invite and aggravate certain epidemic tendencies, and, consequently, we find on examination of the map indicating the localities where cerebro-spinal meningitis has prevailed, that the largest proportion is to be found where these conditions obtain. In wards where the population is very dense, and live under these unfavorable influences, there have been many more cases than in other wards with better hygienic surroundings."

There is no evidence of the propagation of the disease by contagion or infection. Persons ill of it were constantly removed to other and healthy localities, and yet careful investigation shows no case following exposure in individuals who were in direct contact with the sick.

The largest number of cases occurred as single ones in separate localities. In houses in which there was more than one case, they were, as a rule, limited to one family, or occasionally to two living in the same dwelling, or on the same floor, and exposed to the same ætic factors. Several persons were attacked when away from their homes; in some of these, the local influence could be clearly made out, and such observations are, moreover, valuable by showing a period of incubation, notwithstanding the usual absence of percursory symptoms, as before stated, and the suddenness of the seizure.

